# Motoric Cognitive Risk Syndrome: Symptoms, Pathology, Diagnosis, and Recovery

**DOI:** 10.3389/fnagi.2021.728799

**Published:** 2022-02-02

**Authors:** Ke Xiang, Yin Liu, Li Sun

**Affiliations:** Dizziness Clinic, Jilin Provincial Academy of Chinese Medicine Sciences, Changchun, China

**Keywords:** pre-dementia, gait, vascular, test performance, treatment

## Abstract

The motoric cognitive risk (MCR) syndrome is a pre-dementia condition, marked by the enhanced risk for Alzheimer's disease (AD) and vascular dementia, together with falls, disability, and abnormal movements. The research studies revealed the distinct neurological and non-neurological clinical gait irregularities during dementia and accelerated functional decline, such as postural and balance impairments, memory loss, cognitive failure, and metabolic dysfunctions. The disabling characteristics of MCR comprise altered afferent sensory and efferent motor responses, together with disrupted visual, vestibular, and proprioceptive components. The pathological basis of MCR relates with the frontal lacunar infarcts, white matter hyperintensity (WMH), gray matter atrophy in the pre-motor and pre-frontal cortex, abnormal cholinergic functioning, inflammatory responses, and genetic factors. Further, cerebrovascular lesions and cardiovascular disorders exacerbate the disease pathology. The diagnosis of MCR is carried out through neuropsychological tests, biomarker assays, imaging studies, questionnaire-based evaluation, and motor function tests, including walking speed, dual-task gait tests, and ambulation ability. Recovery from MCR may include cognitive, physical, and social activities, exercise, diet, nutritional supplements, symptomatic drug treatment, and lifestyle habits that restrict the disease progression. Psychotherapeutic counseling, anti-depressants, and vitamins may support motor and cognitive improvement, primarily through the restorative pathways. However, an in-depth understanding of the association of immobility, dementia, and cognitive stress with MCR requires additional clinical and pre-clinical studies. They may have a significant contribution in reducing MCR syndrome and the risk for dementia. Overall, the current review informs the vital connection between gait performance and cognition in MCR and highlights the usefulness of future research in the discernment and treatment of dementiating illness.

## Introduction

The world geriatric population shows an unprecedented and rapid increase, with a >8.5% aged about 65 years and above. Dementia is a common problem among elderly individuals, and the statistics claimed that ~50 million people had been living with dementia in 2019, with a predicted rise of over 150 million in 2050 (Kamoga et al., 2019; Vellani, 2019). In fact, abnormal aging bears immeasurable adverse effects, which appears as a key burden on the patients themselves, families, government, and the health system and economy of the country (2001, 2012). Hence, the rising size of the aging population has stirred a need for exploring and identifying the cause, symptoms, pathogenesis, and pathology of age-related disorders (Ballard et al., 2011; Borland et al., 2020; Force et al., 2020).

A cohort-based study also indicated that more than 17% of the elderly, aged 65 years and above, suffered from self-perceived cognitive abnormalities, while <8% had dementia (Graham et al., 1997; Force et al., 2020). An epidemiological survey revealed that 35% of the population aged 70 years and above showed gait impairments (Verghese et al., 2006). It has also been reported from the Canadian Study of Health and Aging that gait disorder, frailty, posture impairments, and parkinsonism (based on stiffness, weakness, resting tremor, and slowness of movement, better known as bradykinesia) were common co-occurrences with a cognitive decline for over 50% of the aging population, which resulted from the pathological conditions (Camicioli et al., 2007).

The normal aging process involves changes in the sensory, motor, and cognitive performances, with a marked reduction in the walking speed and motor activities (Puig et al., 1988). An abnormal gait and postural impairments precede the cognitive dysfunctions and are termed as early makers and high-risk clinical syndromes of dementia, particularly vascular dementia (VaD) (Verghese et al., 2002; Montero-Odasso and Hachinski, 2014). A slow gait followed by memory decline, or the co-existence of the two, had been initially considered as the obvious geriatric symptoms, which later had been identified as a common pathology (Parihar et al., 2013). Relevantly, the motor cognitive risk (MCR) comprised this transitional state among the typical aging, dementia, and mild cognitive impairment (MCI) (Verghese et al., 2013b). It has been reported that the MCR accounts for more than 6.5% of the population aged between 60 and 100 years, with Parkinson's disease (PD), ischemic and cerebral stroke, depression, and metabolic disorders as key contributors (Verghese et al., 2014a). A collective data from the seventeen countries comprising 26,802 elderly people (age range: 60–114 years) demonstrated ~9.7% cases of MCR. People above 75 years of age demonstrated a significantly higher motoric and cognitive impairment, together with dementia, irrespective of sex (Allali et al., 2016). Additionally, the MCR cases in six low- or middle-income countries were ~5.3–15.5%, respectively (Verghese et al., 2014a). The prevalence of MCR has also been found to be higher in persons aged 75 years and older, at par with the greater prevalence of cognitive complaints and dementia in this age segment (Verghese et al., 2014a).

An assessment of MCR is a relatively less complicated method, and hence, may serve as an important tool to predict and help the evolving public health-related disciplines that may control dementia and related pathologies. An added advantage observed with MCR diagnosis is that it may be considered objectively, based on the reduced walking velocity, independent of the fundamental cause (Meiner et al., 2020). The psychometric assessment showed that MCR appeared as a greater predictor for dementia rather than cognitive and motoric lacunae (Allali et al., 2016). These findings helped in the development of novel concepts in the secondary prevention of dementia and in framing the public health-related policies.

In the current review, we will focus on the symptoms shared by dementia, gait impairment, and MCR, including their common pathological processes and associated risk factors. We will also include the pharmacological and non-pharmacological treatments and the procedures for the management of MCR and related dysfunctions.

## Symptoms

**Figure 1:**
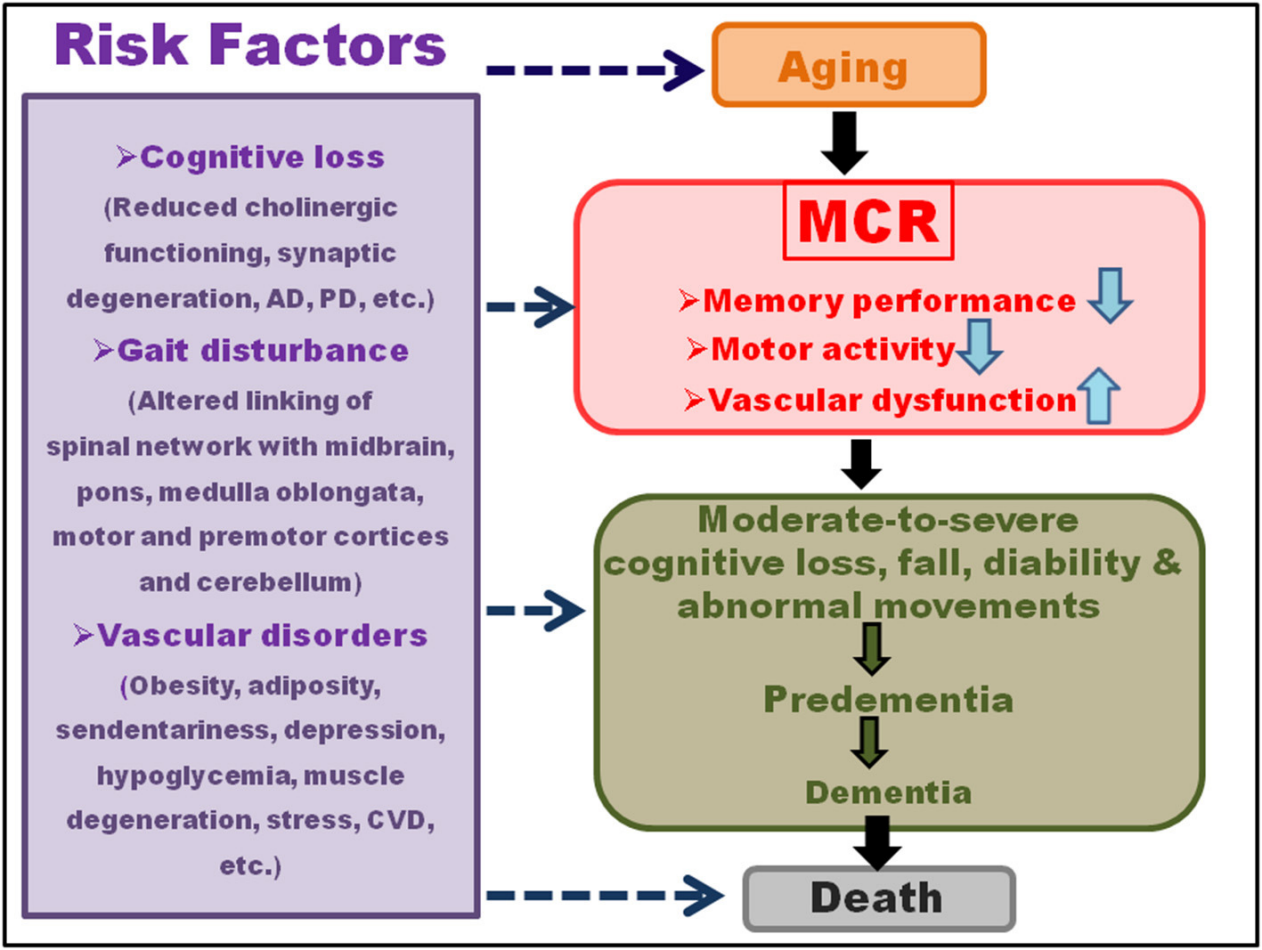
Symptoms and risk factors of motoric cognitive risk (MCR). Motoric cognitive risk, characterized by the reduced memory and gait performances, is an intermediate condition between aging and dementia. The cognitive damage may involve reduced cholinergic functioning, synaptic degeneration, Alzheimer's disease (AD), Parkinson's disease (PD), etc. Gait disturbances may result from altered linking of spinal network with midbrain, pons, medulla oblongata, motor and pre-motor cortices, and cerebellum. The vascular disorders may comprise obesity, adiposity, sendentariness, depression, hypoglycemia, muscle degeneration, stress, cardiovascular disease (CVD), etc. A moderate-to-severe cognitive loss, falls, disability, and abnormal movements during aging may lead to pre-dementia and then dementia, and culminating into death within a short span of MCR diagnosis. The risk factors for this progression range from the vascular, muscular, cognitive to mental disorders.

The MCR is an intermediate condition amid aging and dementia (Figure 1), which shares an analogy with MCI. However, while MCI predominantly deals with cognitive aberrations, the MCR may be categorized as non-amnestic MCI, having a marked link with VaD (Ayers et al., 2020). The patients with MCR bear 3- and 12-fold chances of developing dementia and vascular aberrations, respectively. The MCR also serves as an important predictor for screening the future fall, mobility disability, and mortality (Chhetri et al., 2017) (Table 1). A significant association was reported [778 participants from the Epidemiologie de l'Osteoporose (EPIDOS)] between the physical and cognitive infirmities for MCR, resulting in an augmented risk for short-term (i.e., 5-year), medium-term, and long-term mortalities, where slow walking speed acted as the key predictor for the MCR associated symptoms (Beauchet et al., 2019a).

**Table 1:** Motor, cognitive and vascular impairments in MCR.

**MCR syndrome**	**Symptoms**	**Pathology**	**Diagnosis**	**Recovery**
Motor	Altered gait, imbalance, fall, muscle degeneration, and frontal ataxia (Verghese et al., 2014b; Chhetri et al., 2017)	Altered skeletal musculature and joints, loss in the vastus lateralis and muscle strength, deregulated cholinergic system, altered frontal subcortical circuits, aberrant primary motor cortex at the frontal lobe of the posterior precentral gyrus, peripheral nerves, and neuromuscular junctions that affect synaptic impulses, deregulated cortical motor centers, vestibular and somatosensory systems, pathological features of AD and PD, cerebral Aβ (mainly in the frontal, striatal, temporal, parietal, anterior cingulate, precuneus and occipital cortices) and p-tau deposition, together with neuroinflammation (Devos et al., 2010; Yang et al., 2016; Montero-Odasso et al., 2017; Nadkarni et al., 2017; Ferrazzoli et al., 2018; Mackinnon, 2018; Shen et al., 2020; Van Der Leeuw et al., 2020; El-Baba and Schury, 2021)	Assessment of psychomotor speed, MRI, brain imaging, total physical performance, Computerized Tomography, walking test, chair stands, and tandem stand (Wicherts et al., 2007; Sohl et al., 2013; Walshe et al., 2015; Blumen et al., 2019)	Executive function, cognitive training, practiced dual-task condition, Enhanced physical activity, cognitive enhancers, such as cholinesterase inhibitors (galantamine) and the N-methyl-D-aspartate (NMDA) receptor antagonist, memantine (Assal et al., 2008; Montero-Odasso et al., 2009; Silsupadol et al., 2009b; Rebok et al., 2014; Cavalcante et al., 2018)
Cognition	Dementia, mood disorder, mental disturbances, psychiatric problems, episodic memory loss, depression, stress, anxiousness, worries, fear, loneliness, malice, mood disorders, and neuroticism (Verghese et al., 2014b; Chhetri et al., 2017; Ayers et al., 2020)	The deregulated cholinergic system, neurodegenerative frontal and subcortical regions, pathological features of AD and PD, gray matter atrophy, altered neurotrophin levels (Devos et al., 2010; Rovio et al., 2010; Dada et al., 2014; Beauchet et al., 2016; Hsueh et al., 2018; Blumen et al., 2019)	Frontal Assessment Battery, brain imaging, biomarker monitoring, neuropsychological tasks for memory, concentration, depression, assessing structural and functional pathways of dementia and associated anomalies (Beauchet et al., 2016; Blumen et al., 2019)	Enhanced neuroplasticity, cognitive training, cognitive enhancers, such as cholinesterase inhibitors (galantamine) and the N-methyl-D-aspartate (NMDA) receptor antagonist, memantine (Assal et al., 2008; Montero-Odasso et al., 2009; Cai et al., 2014; Rebok et al., 2014)
Vascular	Cardiac problems, hypertension, obesity, adiposity, sedentariness, stress, diffuse vascular lesions (Verghese et al., 2014b; Wardlaw et al., 2015; Ceide et al., 2018; Ghaznawi et al., 2021)	Periventricular WMH, pulmonary arterial hypertension, carotid atherosclerosis, decreased supratentorial white matter volume, *FTO* gene polymorphism (Schmahmann et al., 2008; Schuff et al., 2009; Ho et al., 2010; Keller et al., 2011; Reitz et al., 2012; Verghese et al., 2013a; Caselli et al., 2015; Smith et al., 2015; Wardlaw et al., 2015; Chen et al., 2017; Ghaznawi et al., 2021)	FLAIR and MRI (Wardlaw et al., 2015; Ghaznawi et al., 2021)	Exercise and a vibrant lifestyle and cerebrovascular drugs (Gothe et al., 2012; Cavalcante et al., 2018)

The MCR involved changes in the intricate interaction of sensory, cognitive, and motor activities that are also affected in the initial stages of dementia (Chhetri et al., 2017). Memory performance, gait, and vascular factors showed a strong association with aging, and the clinical and neurological gait disorders generally preceded cognitive impairment and vascular dementia. The functional changes in gait were the key indicators of non-Alzheimer's dementia in the Bronx Aging Study, and a reduction in the gait velocity signified cognition loss as per the Einstein Aging Study (EAS), marked by irregular frontal gait that failed to match the parkinsonian gait (Verghese et al., 2002). The EAS and Bronx County study assessed the individuals through the computerized walkway, associated with embedded pressure sensors that measured the gait speed (centimeter/second) at a regular pace of walking, using the efficient and authentic GAITRite system. The metabolic disorders, such as cardiac problems, hypertension, rheumatoid, neurological syndromes, cerebral disorders, demographic factors, and illness burden, together with neuropsychological tests, tests for memory, concentration, and depression, were taken into consideration (Ceide et al., 2018). The studies in the elderly persons above 65 years of age in the United States and Europe demonstrated 70% greater chances of mortality, co-existent with MCR (Dewey and Saz, 2001; Sachs et al., 2011; Perna et al., 2015). The mortality risk was prominent within 2 years of MCR diagnosis, and at par with that of moderate-to-severe cognitive loss and predementia (Wilson et al., 2009; Park et al., 2014), with a higher rate for men than women (Kelman et al., 1994; Perna et al., 2015). The typical features together with the MCR-induced mortality included geriatric syndromes, such as dementia, mood disorder, mental disturbances, incontinence, imbalance, dizziness, falls, medication mismanagement, and psychiatric problems. The Cardiovascular Health Study cohort predicted increased mortality in the cases with combined cognitive and gait impairments, showing structural, functional, or pathological alterations in common areas of the brain, along with the upregulated inflammatory markers, particularly interleukin-6 (IL-6), even in the patients with or without signs of dementia (Rosano et al., 2008; Nagga et al., 2014). The major risk factors of importance for MCR, included cognitive loss, gait disturbance, vascular disorders, obesity, adiposity, sedentariness, stress, depression, hypoglycemia, muscle degeneration, and cardiovascular diseases (Figure 1; Verghese et al., 2014b).

The risk factor for MCR increases with age, having a higher prevalence (10–11%) for the age group ≥75 years, relative to 8–9% for 60–74 years, as reported in a study comprising of 26,000 individuals (Verghese et al., 2014a). On the contrary, several studies showed that the incidence of MCR was independent of age (Larner, 2015; Maguire et al., 2018; Shim et al., 2020). Regarding sex-dependent MCR, contradictory reports have been obtained. While a majority of the studies demonstrated a higher prevalence among the women, linked with physical frailty and age (45–54 and 65–74 years), the dissimilar findings were reported for the population of other ages (Beauchet et al., 2019b; Lau et al., 2019). Notably, the frequency of MCR was lower in the more educated strata, probably owing to the education-induced higher brain cognitive reserves (Sharp and Gatz, 2011; Verghese et al., 2014b). An association link between the increased sedentary lifestyle and MCR has also been reported, observed with increased cardiovascular disorders, inflammation, and reduced immune functioning and cerebral neurotrophin levels (Rovio et al., 2010; Dada et al., 2014; Hsueh et al., 2018).

The MCR showed a distinct link with energetic behavior, meticulousness, depression, stress, anxiousness, worries, fear, loneliness, malice, mood disorders, and neuroticism. An inverse link has been reported among the MCR, candidness, and meticulousness, whereas a direct relationship existed among the MCR, mental disorders, and loss in episodic memory, and the cognitive regulation of behavior and working memory (Ayers et al., 2020). Here, the five-factor model, i.e., neuroticism, extraversion, openness, agreeableness, and conscientiousness were the personality traits controlling the MCR and pre-dementia syndrome (Mccrae and John, 1992; Ayers et al., 2020) (Table 1).

## Pathology

**Figure 2:**
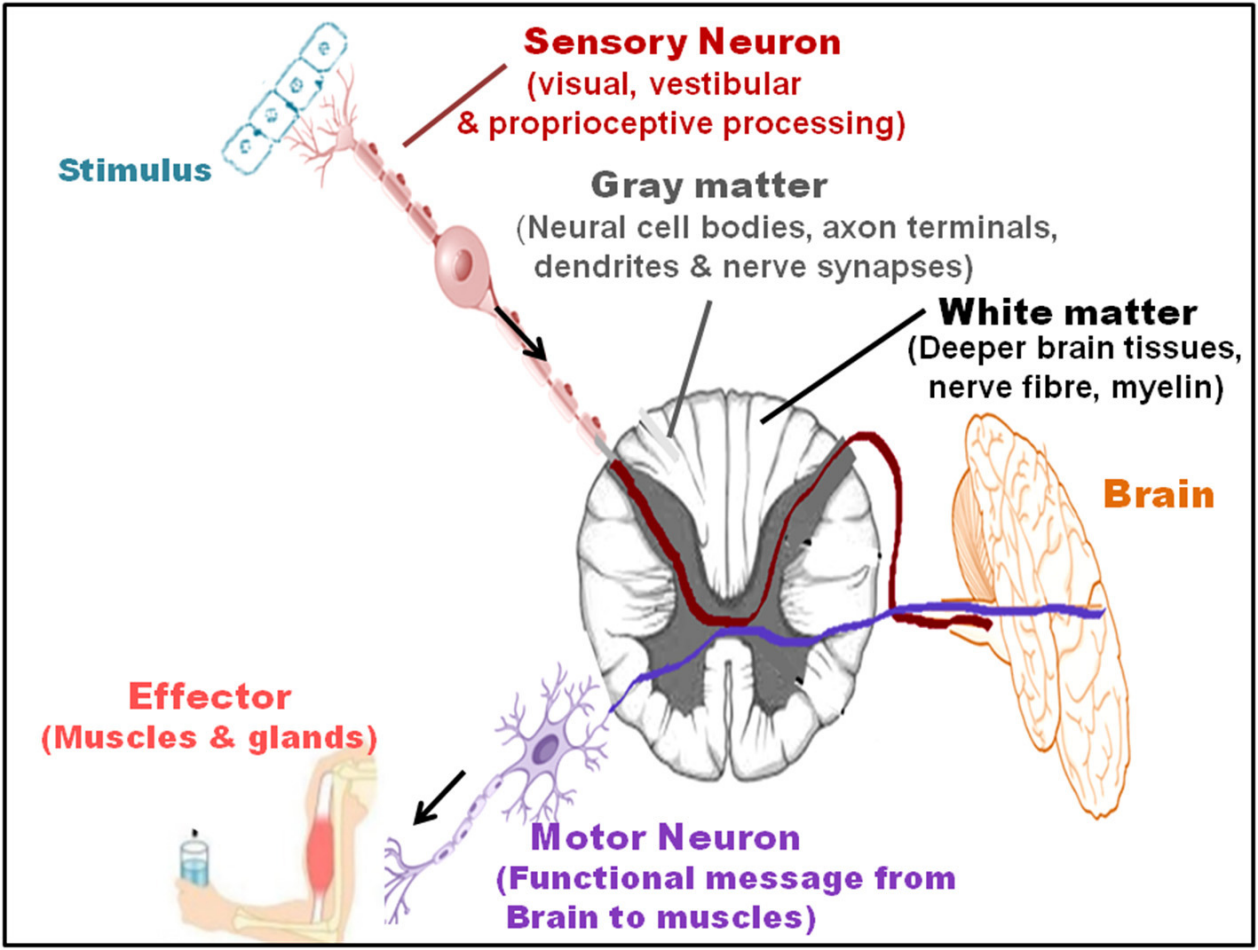
Sensory and motor pathways and interaction with the brain. The combination of sensory (visual, vestibular, and proprioceptive processing), motor, and central processing sustains the functions restricting MCR. The sensory neurons transmit impulses, via their receptors, to the gray matter (comprised of neural cell bodies, axon terminals, dendrites, and nerve synapses), and white matter (located in the deeper brain tissues and includes nerves fiber and myelin sheath) of the brain. Motor neurons that primary sends the functional messages from the brain to the muscles further transmit impulses to the effector, generating a response.

The combination of sensory (constituting visual, vestibular, and proprioceptive components), motor and central processing sustain motor, and cognitive functioning plays a key role in maintaining the stationary and dynamic balance (Wiesmeier et al., 2017). A shift in the coordination of the three systems (Figure 2) leads to marked imbalance and accelerated reduction in the related functions during aging. The skeletal musculature and joints have a significant impact in controlling the motor apparatus and balance, and a loss in the vastus lateralis and muscle strength (common with increasing age) enhances fall risk, imbalance, and impaired movement (Yang et al., 2016). Moreover, the central nervous system (CNS), which coordinates the overall sensory information, generates signals for motor skills and reflexes and, integrates cognitive and postural controls (Nnodim and Yung, 2015). The cholinergic system also links cognition, balance, and motor output, *via* a regulated acetylcholine level and acetylcholinesterase activity (Devos et al., 2010).

The gait activity is primarily regulated by the frontal subcortical circuits that manage interlinked attention and executive control and plays a key role in coordinating the functions of several neural networks of motor, sensory, and cognitive performances (Ferrazzoli et al., 2018). A normal gait cycle also results from the combined interaction of the spinal network with the midbrain, pons, medulla oblongata, motor and pre-motor cortices, and cerebellum (Takakusaki et al., 2016). The abnormalities in gait activity, which bears a key impact on the quality of life, morbidity, and mortality, have been categorized as neurological and non-neurological types. The neurological aberrations entail a key connection between the focal or diffuse brain injury and the primary motor cortex at the frontal lobe of the posterior precentral gyrus, peripheral nerves, and neuromuscular junctions, affecting the synaptic impulses (El-Baba and Schury, 2021). The focal vascular lesions lead to deep cerebral white matter infarcts in the basal ganglia, pons, thalamus, basal ganglia, or brainstem (Hegde et al., 2011). On the other hand, the diffuse vascular lesions involve periventricular white matter hyperintensity (WMH) (detected through the high signal intensities on fluid attenuated inversion recovery (FLAIR) magnetic resonance images) and ischemic changes (Wardlaw et al., 2015; Ghaznawi et al., 2021), secondary to acute vasoreactivity for the patients with pulmonary arterial hypertension. WMH comprises the reduced cerebral blood flow, oxygen consumption, and oxygen metabolism in the frontal, temporal, and parietal white matter of the cerebral cortex (Schmahmann et al., 2008; Schuff et al., 2009). It also engages altered primary excitatory thalamocortical signaling and anterior corticospinal tract (Thompson, 2001).

The gait abnormalities involve aberration in the neural pathways that link cortical motor centers (that regulate planning, control, and voluntary movements) with the neuromuscular system (comprised of motor neurons, sensory neurons, and skeletal muscle fibers) (Verghese et al., 2002). The neurological gait dysfunctions, including the ones belonging to unsteady, frontal, and hemiparetic gait categories, as well as gait apraxia of frontal origins, serve as the key predictors for VaD, mainly of the non-Alzheimer's type (Verghese et al., 2002). In addition, the neurological factors include sensory ataxia, polyneuropathy, parkinsonism, Huntington's disease, normal pressure hydrocephalus, Binswanger's disease, white matter-based small vessel dementia, and documented arterial hypertension. Additionally, cerebral weakness, reduced muscle strength, Charcot–Marie–Tooth disease that causes peripheral nerve damage, cerebellar degeneration, genetically inherited spinal muscular atrophy, sciatic nerve dystrophy, and microvascular white-matter disease induce gait disabilities. Altered sodium-potassium and magnesium imbalance, vitamin deficiency rheumatoid arthritis, osteoarthritis and joint inflammation, stiffness and swelling affect the neurological and musculoskeletal functions, impeding the gait performance (Ayers and Verghese, 2014).

The gait impairments may be categorized into the low-, middle-, and high-levels (Nutt, 2001). The low-level gait abnormality entails aberration in the main sensory system that comprises the visual, vestibular, and somatosensory systems participating in the postural control and balance (Mackinnon, 2018). The middle-level disorders induce muscle stiffening and contraction, involving reduced fluid mobility from cervical spondylotic myelopathy, cerebrovascular problems, Parkinsonism, and non-progressive congenital ataxia. A reduced gait velocity has been considered as a clinical biomarker for high risk of dementia (Cohen and Verghese, 2019). It has also been shown that the gait velocity reduced and stride and stance time enhanced significantly (*p* < 0.01) for the cognitive dysfunctions, during the performance of dual-task (Penko et al., 2018). While the stride time, stance time, and swing time demonstrated an increase during the dual-task, their changes during single-task (ST) walking were moderate or relatively low (Howcroft et al., 2014). Compared with the single task, the dual task participants exhibited a greater loss in the gait and stride length, while the change in the stride velocity was relatively low (Leland et al., 2017). On the other hand, high-level gait disorders include frontal ataxia, psychogenic motor disorder, a disproportionate increase in immobility, and fear of walking (Pirker and Katzenschlager, 2017). In addition, carotid atherosclerosis (marked by systemic global cerebrovascular dysfunction, small-vessel heart diseases, and mixed lesions) leads to memory loss and reduced visual acuity, flexible thinking, and mental skills. Together with this, decreased simple motor ability and motor/processing speed have been reported in these patients (Chen et al., 2017).

A combination of the CNS and peripheral nervous system functioning induces normal gait, which comprises a stance phase and swing phase, with altered hip flexion of varied duration that regulates the gait speed. These regions receive inputs from the cortical primary somatosensory area and orient them into specific behavioral objectives (Pirker and Katzenschlager, 2017). The dysfunctional and neurodegenerative frontal and subcortical regions are common pathological features of aging, which have a vital impact on motor control, balance, mood, cognition, etc. (Montero-Odasso et al., 2017). The cardiovascular risk factors also affect these features, linked with leukoaraiosis, focal vascular lesions, lacunar infarcts, and cerebral microbleeds in the periventricular white matter that supports gait and balance (Marek et al., 2018). Hypertension, diabetes, hyperlipidemia, as well as vascular structural aberrations in the subcortical myelinated white matter and gray matter of the CNS, induce motor coordination abnormalities (Pantoni and Garcia, 1997; Leaper et al., 2001; Pugh and Lipsitz, 2002; Rosano et al., 2005; Shin et al., 2021). The Rotterdam Scan Study for cerebral white matter lesions in the elderly population demonstrated a strong link between the periventricular white matter lesions (that reduced the mental processing speed and fluid intelligence measures of thinking ability and rationality), decreased the psychomotor speed, and showed a relatively weaker association with memory loss (De Groot et al., 2000). These results from acute vasoreactivity and cerebral small vessel disease that adversely affect the pulmonary arteries, arterioles, venules, and brain capillaries and induce small subcortical infarcts (Li Q. et al., 2018). The GAIT study involving 171 MCR and non-MCR participants showed a reduced cortical gray matter volume, particularly in the dorsolateral segments of the pre-motor and pre-frontal cortices. Additionally, the white matter ischemic changes in coronary microvascular disease increased the MCR symptoms. The study emphasized the participation of the pre-fontal cortex in MCR at the preliminary stage of dementia, and the features were quite similar to that of Lewy body dementia or dysexecutive deficits of Alzheimer's disease (AD). Additionally, the study demonstrated the involvement of (a) brain region controlling motor movements related to the kinematic and dynamic parameters, and (b) both the cognition and motor functioning that corresponded with the cerebral cortex covering the frontal lobe and dorsal-lateral segment. A reduced Frontal Assessment Battery, that distinguished between dementias of frontal dysexecutive phenotype and AD, suggested the involvement of the dorsolateral pre-frontal cortex in MCR (Boban et al., 2012).

Magnetic resonance imaging showed that the temporal and spatial gait parameters had a distinct connection with the lacunar infarcts and subcortical brain atrophy, with reduced gait velocity, variability in stride length, as well as altered left and right double limb stance (Rosano et al., 2006). MRI revealed changes in signal intensity in the thalamocortical fibers (that transmit sensory and motor information) and corticospinal pyramidal tracts. These findings indicated interference in the long loop reflexes, essential for steady stride and footing, and a reduction in step length variability, which correlated with a cerebral infraction, arterial vaso-reactivity, and WMH (Rosano et al., 2007). The frontal lacunar infarcts appeared as the key cause for MCR through the interruption of frontal neural networks that regulate cognition and motor functions. The association of frontal lacunar infarcts was stronger for reduced gait speed rather than the cognitive decline in MCR. While a Multicenter European study showed a connection between the sharply reduced working memory, flexible thinking, and self-control with that of psychomotor speed (Jokinen et al., 2011), the lacunar infarcts demonstrated a prominent link with the higher-order cognitive tasks (Baune et al., 2009). Silent brain infarcts, devoid of any apparent clinical phenotype, are related to the slow gait and decreased supratentorial white matter volume (Smith et al., 2015). The participation of the frontal lobe in the motor coordination drew support from functional near infra-red spectroscopy (Holtzer et al., 2011) and functional MRI techniques (Blumen et al., 2014), where the non-amnestic cognitive activities, such as mental skills of working memory, flexible thinking, and attention had a strong association with gait activity (Blumen et al., 2014). It has even been found that the frontal lacunar infarcts rather than cerebral microbleeds held a stronger relationship with dementia (Patel et al., 2013). Moreover, the MRI studies revealed a reduced volume of the global and regional gray matter, particularly in the pre-motor, insular and pre-frontal cortices, as well as the dorsolateral segment of the pre-frontal cortex in the MCR individuals, in contrast to the MCI and other non-MCR cases. This had a strong association with gray matter atrophy, having an adverse effect on motor planning and movement control (Beauchet et al., 2016; Blumen et al., 2019). On the other hand, the MRI modalities revealed moderate levels of degeneration in the medial temporal lobe, primary olfactory cortex, basal forebrain cholinergic system, along with frontal and temporal lobe loss in the patients with MCI (Chandra et al., 2019).

The frailty phenotype involves multiple underlying dimensions, with reduced ability to tolerate stress and enhanced vulnerability to cognitive loss, vascular dementia, AD-like pathology, and MCR, often resulting in severe ailments and death. The cerebral amyloid beta (Aβ) (mainly in the frontal, striatal, temporal, parietal, anterior cingulate, precuneus, and occipital cortices) and p-tau deposition that induces cognitive impairments to contribute to frailty and an adverse impact on the gait speed (Nadkarni et al., 2017; Shen et al., 2020). The connectivity of Aβ here associated with the executive function capabilities and Apolipoprotein E epsilon 4 (APOE ε4) carrier status (pre-dominantly in the anterior brain regions) that regulate the cortical control of gait and also tau hyperphosphorylation (Verghese et al., 2011; Nadkarni et al., 2017). Besides, irrespective of the APOE status, a decreased gait speed related to the Aβ deposition in the posterior and anterior putamen (that shares a direct interaction with the pre-motor cortex, anterior cingulate, and sensorimotor cortex) and occipital cortex (linked with the visual cortex) (Del Campo et al., 2016; Semba et al., 2020).

An increased link between the MCR and frailty has been identified through inflammation. MCR had a connection with single-nucleotide polymorphism (SNP) in the promoter and 3′ untranslated regions (3′ UTR) of the anti-inflammatory IL-10 gene [studied in an Ashkenazi Jewish population (Sathyan et al., 2017)]. Here, the homozygous deletion of IL-10 in mice generated a phenotype that matched with human frailty, reduced vascular relaxation, and endothelial dysfunction. Moreover, IL-10 polymorphism promoted dementia through the intermediate involvement of MCR (Sathyan et al., 2017). Additionally, MCR had a vital link with SNPs in the regulatory region of the 9p21-23 region that relates with the age-dependent complex diseases and frailty (Sathyan et al., 2018). The social vulnerability index comprises factors, such as economic and sociological measures, enlightenment status, education, approach to healthcare, social assistance, and mental condition and disorders, such as depression, apathy, and loneliness (Andrew et al., 2008; Armstrong et al., 2015; Donovan et al., 2016; Lozupone et al., 2018) also coupled with frailty and MCR (Sathyan et al., 2019).

An increased expression of the inflammatory markers during the aging bears a key link with aberrations in cytokine levels, particularly IL-6 and tumor necrosis factor alpha, as well as C-reactive proteins (Ng et al., 2018). Chronic inflammation is an inducer of functional loss and infirmity, associated with the reduction in muscle mass, endurance, flexibility, power, and speed. This causes muscle atrophy and an impaired muscle integrity and function (Rantanen et al., 1999; Visser et al., 2002). The increased serum cytokine levels serve as prognostic markers for incident mobility limitation, measured by trouble or failure in walking and climbing (Penninx et al., 2004). The physical functions, determined by the walking speed, chair stand test, and the standing balance test showed a significant decline, together with the reduction in muscle and grip strength (determined applying a hand-held dynamometer), which is strongly related to the enhanced inflammation (Cesari et al., 2004). The epidemiological studies in the elderly volunteers even indicated a significant link between the upregulated serum IL-6 levels and reduced memory functions of Encoding and Recall (Elderkin-Thompson et al., 2012).

The cognition, motor function, and pain were correlated, as has been reported from the cohort-based studies in the older adults, following adjustment for age, race, sex, and socioeconomic conditions (Van Der Leeuw et al., 2016). The severity of pain, particularly of the continuing moderate to acute category, appeared proportional to dementia and cognitive decline. Typically, the insular- and pre-frontal-cortex, that govern the afferent processing mechanisms and cognition-related modulatory systems, regulated MCR as well as pain. Additionally, an increased inflammation and the serum C-Reactive proteins appear responsible for pain sensitivity as well as altered cognitive and gait phenotype (Van Der Leeuw et al., 2020). The motor dysfunctions, such as weakness, slow movement, tremor, axial impairment, and rigidity related with cognitive decline and also associate with dopaminergic neurodegeneration in PD (2018). A combination of aberrant righting reflexes, gait disturbances, visuospatial acuity that controls the movement, path integration skills, spatial orientation, and navigation also contribute to the motor impairments. In reality, a marked link has been detected between the bradykinesia (the key manifestation of PD) and loss in cognitive flexibility, rationality, episodic memory, contextual information, and axial features, with dopaminergic neurodegeneration (Ataullah and De Jesus, 2021). The cohort-based studies indicated a very strong connection between MCR and AD, owing to the greater mixed brain pathology (Schneider et al., 2007; Bennett et al., 2012a,b). A high association of vascular diseases and strokes was reported with MCR, with a relatively lower link with PD (Verghese et al., 2014a). Furthermore, the cardiovascular diseases and hypertension served as predictors for MCR (Verghese et al., 2014a).

Poor nutrition increased MCR, where vitamin D deficiency had a contributory role in reducing calcium absorption and increasing bone fragility and chances of osteoporosis. The decreased serum 25(OH)D levels (below 20 ng/ml) related with total physical performance, assessed through walking test, chair stands, and tandem stand (Wicherts et al., 2007; Sohl et al., 2013). Vitamin D deficiency increased the chances of cognitive loss, pre-dominantly in the aged people. Additionally, the deficits in vitamin C, B12, and folates triggered neuroinflammation and a cascade of inflammatory markers, particularly IL-6, reduced metabolic rate, and increased chances of motor as well as cognitive impairments (Chrysohoou et al., 2004; Inzitari et al., 2011).

Polypharmacy, which involves the usage of five or more regularly scheduled medications, has also been considered as a modifiable risk factor for MCR. A study in a total of 1,119 adults (≥65 years) in a cross-sectional cohort of Community-based Health and Retirement Study showed that polypharmacy, that marked for the medical co-morbidities and complexities, related with decline in the gait velocity and cognitive loss as compared with the ones without polypharmacy (George and Verghese, 2020). Polypharmacy appended the chances of adverse drug events and interactions, intentional and non-intentional medical adherence, and reduced treatment effectiveness that increased MCR through the metabolic and structural alterations in the brain (George and Verghese, 2020).

Polygenic score (PGS), structured on SNPs derived from the genome-wide association studies (GWAS) demonstrated a link among dementia, cognition, and MCR (Sathyan et al., 2019). Genetic pre-disposition for conditions, such as midlife obesity, cerebrovascular disorders, neuroticism, neurodegenerative diseases (PD and AD), sedentary lifestyle, and depressive symptoms increased MCR, whereas, the higher educational entity reduced its chances. A greater Basal Metabolic Index (BMI), mean arterial pressure, and waist measurement (obtained from multicenter studies in aged population of the United States and Japan) (Janssen et al., 2004; Luchsinger et al., 2007; Doi et al., 2015; Mahlknecht et al., 2015) had a significant link with MCR, vascular dementia, and decreased regional and global gray matter volume. Increased BMI altered the default mode network connecting the pre-frontal cortex, posterior cingulate cortex, precuneus, and angular gyrus, obtained from the logistic analysis of PGS. The genetic susceptibility of the obese patients for developing MCR may have been due to the pleiotropic role of SNPs for both obesity and vascular dementia. A typical example is that of the fat and obesity-associated (FTO) polymorphisms in the *FTO* gene (that signifies polygenic obesity) (Ho et al., 2010; Keller et al., 2011; Reitz et al., 2012; Verghese et al., 2013a; Caselli et al., 2015). The APOE ε4 allele that relates with the late-onset AD also showed speedy loss in gait in the older people. For ApoE4 (genotype) carriers, the change in gait velocity served as a predictor for the decreased memory. A multi-country study also showed that the recovery from MCR increased in people lacking the APOE4 allele (Mahlknecht et al., 2015).

## MCR Diagnosis and its Advantages

First, the gait velocity (measured as centimeter/second), and particularly clinical gait evaluation is a precise marker and diagnostic tool for the cognitive decline, geriatric signs, and for predicting overall health status, because immobility paves the way for dementia (Ayers and Verghese, 2014; Callisaya et al., 2016). Falls are also assessed for MCR diagnosis, normally through a questionnaire. Generally, the brain imaging, biomarker monitoring, and neuropsychological tasks for assessing the structural and functional pathways of dementia and associated anomalies are diagnostic tools for dementia (Risacher and Saykin, 2013). MCR is assessed through (a) cognitive status as a benchmark, involving regular memory and cognitive loss on the pre-set questionnaires; (b) reduced gait velocity compared with age and sex; and (c) ability to move around without signs of dementia. The protocol for measuring the gait speed is relatively easily adopted, even by the non-professional persons, without the requirement of expensive tools and procedures. The assessment of motor performance has a significant advantage in the terms of predicting the causes of MCR, such as vascular lesions and disorders, cerebral small vessel diseases, leukoaraiosis and morphological, and the functional abnormalities of the brain that relate with the reduced gait and cognitive decline (Verghese et al., 2014b) (Table 1).

## Recovery

**Figure 3:**
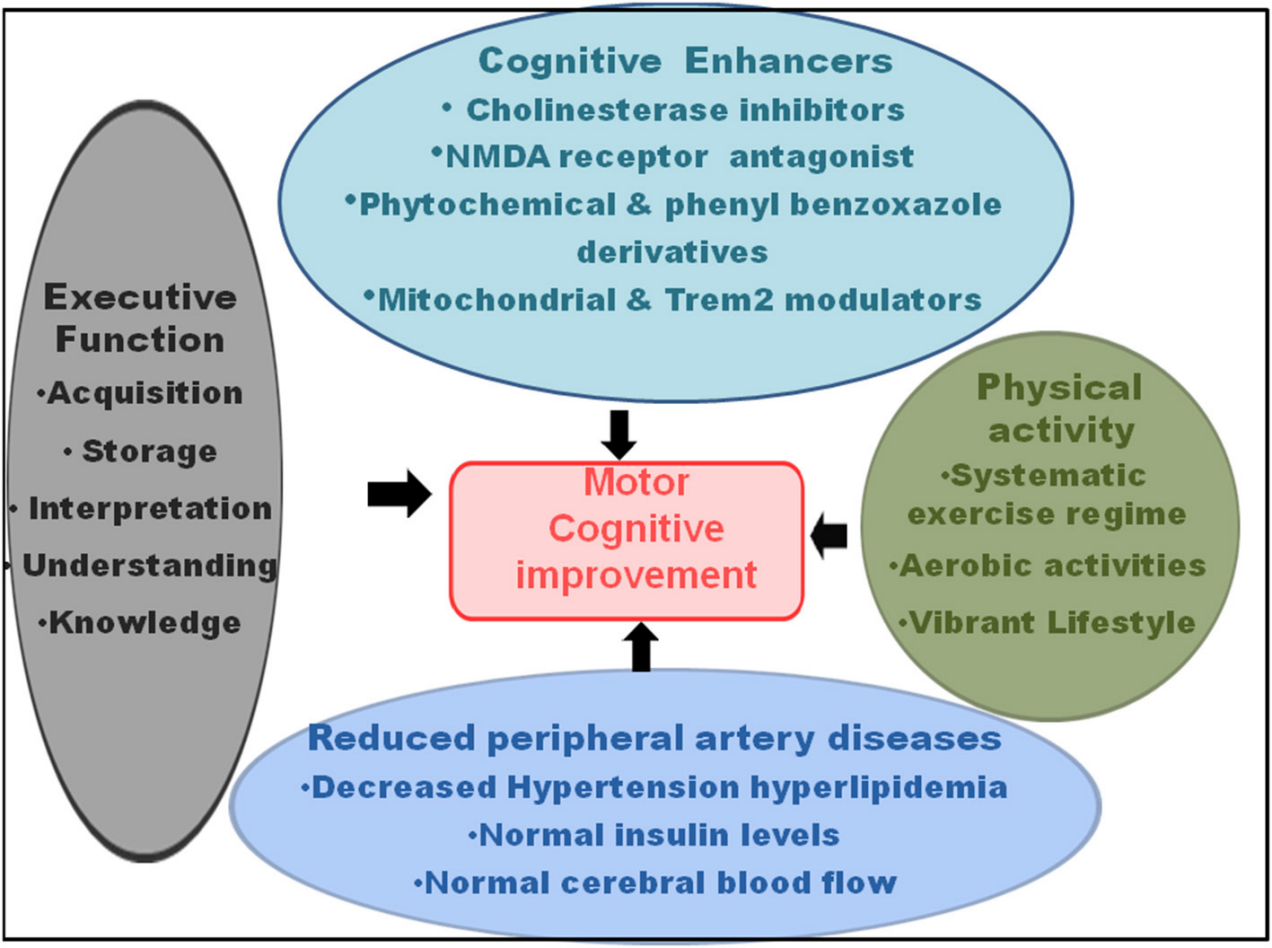
Recovery in the motor-cognitive disorders. The cognitive enhancers, such as cholinesterase inhibitors, N-methyl-D-aspartate (NMDA) receptor antagonists, phytochemical and phenyl benzoxazole derivatives, and mitochondrial and triggering receptor expressed on myeloid cells 2 (Trem2) modulators; physical activity, such as systematic exercise regime, aerobic activities, and vibrant lifestyle; reduced peripheral artery diseases, comprising hypertension, hyperlipidemia, diabetes, and dysregulated cerebral blood flow; and executive functions, such as increased acquisition, information storage, interpretation, understanding, and knowledge improve motor and cognitive functions, and hence, serve as recovery for MCR.

Since a key link exists among the gait, automated motor task, cognitive input, cortical sensory functioning, planning skill, self-awareness, sensory inputs, primary reflexes, and decision-making, mainly in the elderly population, improvement in one influenced the other (Figure 3). Among the above factors, gait and particularly, gait posture, showed a very strong link with the motor task, with a relatively lower necessity of cognitive input (Yogev-Seligmann et al., 2008; Huh et al., 2016; Kabbaligere et al., 2017; Dharmadasa et al., 2018; Kocak et al., 2021). Fine motor coordination also related to cortical sensory functioning (Gottesman et al., 1984), whereas the management of cognition served as a remedy for impairment in the planning, problem solving, executive function, and self-awareness (Tate et al., 2014). The orbitofrontal cortex controlled decision-making and its relation with cognitive functions, particularly working memory (Shadlen and Kiani, 2013; Chick, 2019). Hence, the measures that reduced the gait disorders had a potential impact on the cognitive functions and dementia. Executive function, that involves a battery of cognitive operations of acquisition, storage, interpretation, understanding, and knowledge, along with the information from primary somatosensory area in the ventral-dorsal brain region, had a key influence on the gait and mobility (Demnitz et al., 2017). Increased executive function and attention, that affect the memory performance, also influenced the orientation in physical space and reduced fall risk (even for the ones with fall history) and locomotor problems, even in the clinical settings (Yogev-Seligmann et al., 2008; Smith-Ray et al., 2014). Executive function modifies the processing system in the brain, which enhanced the walking ability and reduced stride-to-stride variability performance in the effortless working-memory task. Testing on the n-back working memory task revealed that for young and aged adults, a simultaneous progression could occur in posture and working memory using retest practice (Yogev-Seligmann et al., 2008; Doumas et al., 2009). Moreover, an enhanced neuroplasticity reduced the age-dependent link between the loss in cognition and gait speed and improved balance (Cai et al., 2014).

The cognitive training that relies on the structural and functional plasticity of the brain plays a key role in sustaining and improving the overall behavior and mental health. Hence, targeting the main cognitive domains that train up the mental skills, such as knowledge acquisition, understanding, application, examination, rationality, assessment, and appraisal capability have a revival impact on the cognitive performance of the brain. The multicentric Advanced Cognitive Training for Independent and Vital Elderly (ACTIVE) study demonstrated that the cognitive training not only has an advantageous effect on the cognitive skills but also on the daily functioning, marked by ease of executing the instrumental activities of daily living, even after 5-year post-cognitive training. The features include reasoning, memory, speed of training, attention, daily problem-solving ability, activities of daily living, such as overall health, reduced delay in functional disability, improved sensory function for object recognition, and sociodemographic (Rebok et al., 2014). The cognitive training also helped in the recovery of auditory sensation, precision, and execution speed (Willis et al., 2006). Notably, together with the increased conscious allocation of cognitive resources, the working memory training (following 10 practice sessions) had a recovery effect on the postural stability and balance, generating a strong foundation for optimal movement (Doumas et al., 2009). The computerized visuospatial cognitive tasks also showed marked recovery in the Timed Up and Go (TUG) that assessed balance. TUG and distracted 10-m walk speed (walking during distraction) compared with the pure gait speed were greater, particularly for the slow walkers. The cognitive training sessions appeared useful for improving the motor coordination disorders, pre-dominantly for the geriatric population that is less eager to undergo a physical exercise training regime (Smith-Ray et al., 2015). CT-spatial navigation training protected from the harmful mobility functioning, particularly following the bed rest. The training caused better or rather positive effect on the walking time, with increased stimulation and sense organ perception under the challenging situations, measured by the dual task walking condition. Here, the improved regular walking, reduced fall, and dual-task effects in the sedentary individuals involved a better recording, processing, responding, and recovery of information. This had an association with the computer-based training of executive functions in the brain regions responsible for mobility. It was observed that the cognitive dual-task training had a recovery effect on the dual-task coordination as a physical outcome (which could improve gross mobility) in the aged persons with mobility restrictions (Walshe et al., 2015). The trainings comprised single-support standing balance (measured by speed, variability, and peak-to-peak excursion) and double-support standing balance [stable platform (SO1), visual surround sway referenced (SO3), and platform sway referenced (SO4) of Gravity Alignment] (Li et al., 2010). The balance problems were much less for practiced dual-task condition, where the performance was best for the participants under variable-priority (VP) instruction, showing more than 50% decrease in the body sway. A marked recovery was observed in the counting backward by 3s task while sitting, and a much faster counting as a cognitive activity following the training. The overall positive effects were far greater for VP training, compared with the ST balance training and dual-task training with fixed-priority (FP) groups (Silsupadol et al., 2009a).

A physical activity that sustains overall fitness and health conditions is an absolute necessity to inhibit the decline in the gross motor skills, fine motor coordination, and body movement involving the skeletal energy expenditure (Figure 3). A systematic exercise regime, comprising a recommended time period of moderate-intensity and vigorous-intensity aerobic activities for few days a week, is necessary for reducing the morbidity and subsequent mortality and for improving the standard of health in the older persons. Additionally, the activities that increase the muscle strength and exercises that raise the balance and prevent fall increase the overall motor activities, together with the improvement in the cognitive functions and vascular diseases (Elsawy and Higgins, 2010).

The gait is not an isolated event, and motor functioning essentially requires the cognitive inputs and neuropsychological impacts. Hence, walking and motor activities result from the integrated interaction of attention control, cognitive flexibility, and a top-down regulation of attention. Supporting this, the randomized clinical data also showed that cognitive remediation program had a positive influence on the gait activity, particularly in the feeble elderly population (Li K. Z. H. et al., 2018). An 8-week cognitive remediation increased the gait velocity when walking at a regular pace in the “walking while taking” condition. Cognitive remediation improved the processing speed of cognitive ability (Verghese et al., 2010). Older persons with better physical ability and activity, regular walking habits, and active lifestyle showed reduced dementia risk and late-life cognitive abilities, along with the increased resistance to the risk factor changes (Abbott et al., 2004). An enhanced physical activity as a recovery strategy for MCR and associated dementia could also be explained from the physiological point of view. Exercise and a vibrant lifestyle improved the vascular health by reducing the peripheral artery diseases, hypertension, blood pressure, irregular lipid profiles, and abnormal cerebral blood flow (Cavalcante et al., 2018). In addition, physical exercise normalized the blood-glucose levels through reduced insulin resistance and glucose intolerance, which are related with the increased memory functions. The physical fitness performances preserved the neuronal morphology and functions, neuronal and vascular interactions, axon formation, synaptic transmission, and capillary extension (Weuve et al., 2004). Nonetheless, even though exercise and activity enhanced physical ability and strength, the task appeared difficult for the physicians. It has been seen that the aged persons have restricted admittance and chances for exercise regimes, with more than 50% leaving the programs within a short span of start. Hence, the cognitive-based mediations have been proposed to upgrade motility, physical adjustability, and flexibility in the elderly. In this context, a meta-analysis study revealed that brain training exercise not only improved the cognitive abilities, but also augmented the physical actions in conditions of complex walking, such as walking while talking or walking while subtracting numbers (Marusic et al., 2018).

In terms of pharmacological intervention (Figure 3), short-term and long-term CNS-active drugs, such as psychotropic, neuroleptics, sedatives, hypnotic, benzodiazepines, non-barbiturate and barbiturate antiepileptic drugs, anticonvulsants, opioids, anti-anxiety, antidepressant, and selective serotonin-reuptake inhibitor drugs increased the risk of fall and fracture (Ensrud et al., 2002; Takkouche et al., 2007; Sterke et al., 2008; Allali and Verghese, 2017). Moreover, the use of digoxin, type IA antiarrhythmic drugs, and diuretics caused falls in the elderly persons (Leipzig et al., 1999; Beauchet et al., 2014a; De Vries et al., 2018). On the other hand, the cognitive enhancers, such as cholinesterase inhibitors (galantamine) and the N-methyl-D-aspartate (NMDA) receptor antagonist, memantine, not only reduced the AD-like symptoms, memory loss, and dementia, but also had an ameliorative effect on the balance, gait and motor dysfunctions, and fall. These interventions had a positive impact on the mental skills, such as working memory, rational thinking, and self-restraint (Assal et al., 2008; Montero-Odasso et al., 2009). However, there has been a controversy among the studies reporting falls, syncope, cardiovascular abnormalities, delirium, and hypotension following the treatment with cholinesterase inhibitors, such as donepezil and memantine (Bordier et al., 2005; Fisher and Davis, 2008). Nonetheless, regarding gait performance, donepezil and memantine demonstrated a beneficial effect by reducing stride time variability (Beauchet et al., 2014a). These effects of the acetylcholinesterase inhibitor, donepezil, and the glutamatergic and dopaminergic modulator, memantine, suggested that the stabilization of neurotransmitter functioning by these drugs played an important role in the gait recovery (Montero-Odasso et al., 2019). Second, the role of memantine could be further explained by its contribution toward regulating glutamatergic transmission from the striatum to gait-related cortical regions, particularly supplementary motor area and dorsolateral pre-frontal cortex (Beauchet et al., 2014a). The acetylcholinesterase inhibitor had the capability of double tasking (walking while counting aloud backward), indicating a coordinated time attention between walking and the counting task. On the other hand, memantine performed better in the single tasking, which measured straight walking at a normal pace (Beauchet et al., 2011, 2014b).

The drugs, such as Biphenyl-3-oxo-1,2,4-triazine linked piperazine derivatives that function as antioxidants and acetylcholinesterase inhibitors, may play a key role in MCR recovery through their efficiency in improving the learning-memory performance and attenuating AD-like pathology (Tripathi et al., 2019). The observations from a study involving *in vivo* and *ex vivo* experiments showed that the efficacy of few phenyl benzoxazole derivatives was comparable to donepezil (Srivastava et al., 2019). Additionally, since mitochondrial homeostasis regulates the synaptic functioning and cognitive ability targeting the mitochondrial dysfunction may reduce AD-like pathology (Rai et al., 2020b). Microglial activation augments AD pathogenesis, and thus, an altered expression of triggering receptor expressed on myeloid cells 2 (Trem2), that acts as a risk factor for AD, may reduce the compaction of Aβ and attenuate the cognitive decline (Rai et al., 2020a). Moreover, several phytochemicals, which act as conventional herbal remedies for AD, may be used to explore their efficacy against MCR (Singh et al., 2021).

## Conclusion and Future Directions

The co-existence of impaired gait and cognitive decline is not only an aging phenomenon, but also involves the overlap of pathological events from the frontal subcortical circuits as well as executive functions. Hence, a distinct discernment of the fundamental cause, biology, and mechanisms of MCR-associated pathology may contribute toward the early recognition of high-risk dementia. It could also offer novel perception into the preventive strategies against the loss in mental abilities and cognition in the geriatric population. An in-depth investigation of both the vascular and non-vascular components could essentially identify the ways through which MCR generates dementia and culminates into death. It may help in developing interventions for the disease and age-associated disabilities, such as reduced intellectual clarity, executive functioning skills, and attentiveness. Since immobility and dementia shows strong linkage, the detailed physiology and mode of one influencing the other require further investigation. Hence, elucidating their association may have a significant contribution for improving the patient and healthcare. Moreover, to understand the cognitive distress in MCR, the comprehensive research and clinical surveys appear essential for uplifting the quality and scope of subjective cognition, often used as a hallmark for MCR detection.

## Author Contributions

KX wrote the manuscript. YL performed literature survey. LS conceived the idea and edited the manuscript. All authors contributed to the article and approved the submitted version.

## Funding

This work was funded by the TCM clinical efficacy evaluation ability improvement project based on the dominant diseases in TCM clinical research base and Jilin Provincial Clinical Research Center for Geriatric Diseases of Traditional Chinese Medicine (YDZJ202102CXJD075).

## Conflict of Interest

The authors declare that the research was conducted in the absence of any commercial or financial relationships that could be construed as a potential conflict of interest.

## Publisher's Note

All claims expressed in this article are solely those of the authors and do not necessarily represent those of their affiliated organizations, or those of the publisher, the editors and the reviewers. Any product that may be evaluated in this article, or claim that may be made by its manufacturer, is not guaranteed or endorsed by the publisher.
